# Efficient Induction of Cytotoxic T Cells by Viral Vector Vaccination Requires STING-Dependent DC Functions

**DOI:** 10.3389/fimmu.2020.01458

**Published:** 2020-07-16

**Authors:** Cornelia Barnowski, Gregor Ciupka, Ronny Tao, Lei Jin, Dirk H. Busch, Sha Tao, Ingo Drexler

**Affiliations:** ^1^Institute for Virology, Düsseldorf University Hospital, Heinrich-Heine-University, Düsseldorf, Germany; ^2^Division of Pulmonary, Critical Care and Sleep Medicine, Department of Medicine, University of Florida, Gainesville, FL, United States; ^3^Institute of Microbiology, Immunology and Hygiene, Technical University Munich, Munich, Germany

**Keywords:** viral vector vaccine, dendritic cells, STING, modified vaccinia virus Ankara MVA, cytotoxic T cells, type I interferon

## Abstract

Modified Vaccinia virus Ankara (MVA) is an attenuated strain of vaccinia virus and currently under investigation as a promising vaccine vector against infectious diseases and cancer. MVA acquired mutations in host range and immunomodulatory genes, rendering the virus deficient for replication in most mammalian cells. MVA has a high safety profile and induces robust immune responses. However, the role of innate immune triggers for the induction of cytotoxic T cell responses after vaccination is incompletely understood. Stimulator of interferon genes (STING) is an adaptor protein which integrates signaling downstream of several DNA sensors and therefore mediates the induction of type I interferons and other cytokines or chemokines in response to various dsDNA viruses. Since the type I interferon response was entirely STING-dependent during MVA infection, we studied the effect of STING on primary and secondary cytotoxic T cell responses and memory T cell formation after MVA vaccination in STING KO mice. Moreover, we analyzed the impact of STING on the maturation of bone marrow-derived dendritic cells (BMDCs) and their functionality as antigen presenting cells for cytotoxic T cells during MVA infection *in vitro*. Our results show that STING has an impact on the antigen processing and presentation capacity of conventionel DCs and played a crucial role for DC maturation and type I interferon production. Importantly, STING was required for the induction of efficient cytotoxic T cell responses *in vivo*, since we observed significantly decreased short-lived effector and effector memory T cell responses after priming in STING KO mice. These findings indicate that STING probably integrates innate immune signaling downstream of different DNA sensors in DCs and shapes the cytotoxic T cell response via the DC maturation phenotype which strongly depends on type I interferons in this infection model. Understanding the detailed functions of innate immune triggers during MVA infection will contribute to the optimized design of MVA-based vaccines.

## Introduction

Vaccinia virus (VACV) is a member of the genus *Orthopoxvirus* and belongs to the family *Poxviridae*. It has a complex structure with a long double-stranded DNA (dsDNA). In contrast to other DNA viruses the replication takes place in the cytoplasm within special organelles called viral factories (1–4). Modified vaccinia virus Ankara (MVA) was derived from the chorioallantois vaccinia virus Ankara (CVA) by serial passaging in chicken embryo fibroblasts (CEFs) (5, 6) causing large deletions and numerous mutations in the viral genome affecting virulence and various immune evasion factors (e.g., factors interacting with host receptors for interferon-γ, interferon-α/β, and CC-chemokines) (7–10). Consequently, the virus is unable to produce infectious particles in most mammalian cells including humans (11, 12). The expression of viral genes is programmed to occur in three consecutive stages: early (118 ORFs), intermediate (53 ORFs), and late (38 ORFs) gene expression (13, 14). Despite the abortive infection, MVA allows for early through late gene expression (15). Due to its high safety profile MVA is currently tested as a recombinant vaccine candidate for various infectious diseases and cancer (16–18).

The innate immune system acts as the first line of defense against invading pathogens involving various cell types such as macrophages, dendritic cells (DCs), neutrophilic granulocytes, natural killer cells, and congenital lymphatic cells (19–21). Viruses activate innate immune system components e.g., by interaction of viral pathogen-associated molecular patterns (PAMPs) with cellular pattern recognition receptors (PRRs) (22). The detection of viral PAMPs via PRRs such as TLRs (toll-like receptors) or CDSs (cytosolic DNA sensors) activates intracellular signaling cascades which amongst others lead to the secretion of type I interferons (e.g., IFN-α, -β), proinflammatory cytokines and chemokines and induce an increased expression of costimulatory molecules such as CD40, CD80, and CD86 e.g., on DCs (23). Particularly for DNA viruses, stimulator of interferon genes (STING) plays a crucial role in this respect. STING is activated after recognition of foreign DNA from bacteria and viruses as well as own cellular DNA that has been released by cell stress or dysfunction (24, 25). The cyclic GMP-AMP synthase (cGAS) is an unusual innate immune sensor as it functions simultaneously as a receptor and as a biosynthetic enzyme (26, 27). The binding of dsDNA to the zinc finger domain of cGAS rearranges the active side of cGAS and enables the synthesis of the secondary messenger cyclic GMP-AMP (cGAMP) (26, 28–31). 2'3'-cGAMP activates the IFN-I signal cascade via the ER-resident homodimer receptor STING. The ability of a single cGAS enzyme to produce multiple cGAMP molecules provides a mechanism that can generate a rapidly amplifying immune response by small amounts of cytosolic DNA (32–34). A conformational change allows the recruitment of TANK-binding kinase 1 (TBK1) which phosphorylates the C-terminal domain of STING and thus leads to further recruitment of the transcription factor IRF3 (35, 36). TBK1 phosphorylates IRF3, IRF3 dimers are formed, and translocated into the nucleus. In the nucleus, IRF3 mediates the transcription of IFN-β and other coregulated genes. It has been recently shown that the expression of type I interferons in MVA-infected bone marrow-derived dendritic cells (BMDC) was completely dependent on STING (37). It should also be noted that STING can activate other signal transduction pathways involving NF-κB-, MAP kinases or STAT6 (38–40) indicating the importance of STING for the induction of potent innate immune responses.

During an infection, naïve cytotoxic CD8^+^ T cells (CTL) are initially activated (primed) by antigen-presenting cells (APCs) in secondary lymphatic organs such as lymph node and spleen (41). The first major cell population in contact with the pathogen in secondary lymphoid organs are CD169^+^ macrophages lining the subcapsular sinus area. Although macrophages may present antigens very effectively, CD8^+^ T effector cells are primarily induced by professional APCs such as dendritic cells. It has been recently demonstrated, that optimal CTL responses induced by MVA vaccines require antigen presentation by infected DC as well as cross-presentation of antigen by non-infected bystander DC with both pathways being executed by distinct DC subsets (42, 43). The accurate killing of infected target cells is mediated by the release of cytotoxic granules (e.g., Granzymes and Perforin) from lysosomal compartments (44, 45). During degranulation, the lysosomal membrane proteins are transiently translocated to the cell surface of the CD8^+^ T-effector cell which thereby become identifiable by the expression of lysosome-associated membrane proteins (LAMP-1 and LAMP-2) (46, 47). The understanding of the molecular interactions between innate and adaptive immunity, particularly with regard to the induction of efficient cytotoxic immune responses, is an important requirement for the development and optimization of MVA-based vaccines.

In the present study, we investigated the role of STING for the induction of an efficient cytotoxic T cell response after viral vector vaccination using recombinant MVA. We demonstrate that STING is crucial for the induction of efficient CD8^+^ T cell responses *in vivo*. Interestingly, mainly the immunodominant CTL response was reduced and only short-lived effector and effector memory T cells were impaired, while long-term memory was unaffected. The proinflammatory cytokine and chemokine response was broadly impaired in infected mice lacking STING. Our *in vitro* findings suggest that the impaired CD8^+^ T cell response in these mice was at least partly due to the abrogated type I interferon response in DCs which resulted in inefficient DC maturation and impaired antigen-processing and presentation capacity.

## Materials and Methods

### Mice and Vaccination

Homozygous MPYS^−/−^ mice (STING KO) and MPYS^+/+^ wildtype littermates (STING WT) were originally obtained from B. Opitz, Charité, Berlin, and have been described elsewhere (48). C57BL/6 mice were purchased from Janvier. Transgenic mice were derived from in-house breeding “Zentrale Einrichtung für Tierforschung und wissenschaftliche Tierschutzaufgaben (ZETT)” under specific pathogen-free conditions following institutional guidelines. Animal experiments have been conducted according to the German Animal Welfare Act (Tierschutzgesetz) and have been approved by the regional authorities (North Rhine-Westphalia State Environment Agency -LUA NRW, Germany). Female mice between 8 and 12 weeks old were used.

### Viruses

Recombinant modified vaccinia virus Ankara (MVA) expressed OVA under the control of the early/late promoter P7.5 or PH5 (49). MVA-P7.5-NP-SIINFEKL-eGFP expressed the influenza A virus nucleoprotein fused to the class I (Kb)-restricted SIINFEKL-peptide epitope of OVA fused to eGFP (50) and MVA-PK1L-OVA expressing OVA under the control of the early promoter PK1L (49). All viruses were purified by two consecutive ultracentrifugation steps through a 36% (wt/vol) sucrose cushion and titrated by using standard methods (51).

### Vaccination

Mice were vaccinated at 8-10 weeks of age by intraperitoneal (i. p.) or intramuscular (i. m.) application of 10^7^ infectious units (IU) MVA-p7.5-OVA in 200 or 100 μl of vaccination buffer (20 mM Tris-HCl, 280 mM NaCl, pH 7.4), respectively. For the i. m. immunization mice were injected with 50 μl virus per leg. Vaccinated mice were either sacrificed on day 7 post-infection (p. i.) or boosted i. p. on day 28 post prime with 10^7^ IU MVA-p7.5-OVA and sacrificed 5 days after the second vaccination. Spleens were harvested and induced CD8^+^ T cell responses analyzed as described below.

### *Ex vivo* T Cell Analysis

Spleens of vaccinated animals were collected and processed into a single-cell suspension by mechanical disruption using a 70 μm cell strainer and a plunger. Erythrocytes were lysed by incubation in lysis buffer (BD Pharm Lyse^TM^) for 1 min at room temperature. Cells were passed through a 70 μm cell strainer and counted using a Neubauer cell counting chamber. Thereafter, 4 × 10^6^ splenocytes were plated at 100 μl per well of a 96-well plate and further incubated with 2 μg/ml of MVA-specific or control peptides and 1 μg/ml brefeldin A (Merck) for 5 h. Peptides were A19_47−56_ (VSLDYINTM), B8_20−27_ (TSYKFESV), K3_6−15_ (YSLPNAGDVI), A3_270−277_ (KSYNYMLL), or D13_118−126_ (NCINNTIAL) derived from MVA and OVA_257−264_ (SIINFEKL) peptide derived from ovalbumin. K3 and D13-derived peptides are H2-D^b^-restricted, all other peptides are H2-K^b^- restricted. All peptides were purchased from Biosynthan (Germany). Beta-galactosidase (β-Gal) peptide was used as negative control as a non-cognate ligand. As an additional control, T cells were stimulated in a non-antigen-specific manner using anti-mouse CD3e antibody (clone 500A2, BD Pharmingen 553238) at 1,25 μg/ml. For the determination of CD107a expression, splenocytes were additionally incubated in the presence of anti-CD107a antibody (eBioscience).

### Generation of BMDCs

Femur and tibiae from 12 to 16 weeks old mice were flushed with M2 medium and erythrocytes were lysed by incubation with 5 ml of diluted BD Pharm Lyse buffer^TM^ for 1 min at room temperature. 5 × 10^6^ bone marrow cells were plated in 10 ml M2 medium (containing 10% heat inactivated FCS, 50 μM 2-mercaptoethanol) and 10% GM-CSF (conditioned medium obtained as supernatant from B16 cells expressing GM-CSF; originally kindly provided by Georg Häcker, Freiburg, Germany) in 10 cm Petri-dishes. On day 3 and 6 cultures were replaced with 10 ml of fresh M2 medium containing 10% GM-CSF, respectively. BMDCs cultures were used for experiments on day 7.

### BMDC Infection

Semiadherent BMDCs were scraped, counted using a Neubauer cell counting chamber and 4 × 10^6^ BMDCs were spun down at 319 xg. After centrifugation, cell pellets were resuspended in 200 μl M2 medium (RPMI 1640 containing 10% heat inactivated FCS, 50 μM 2-mercaptoethanol) and BMDCs were infected at a multiplicity of infection (MOI) of 5 and incubated for 12 h at 37°C and 5% CO_2_. In the first hour of infection BMDCs were softly shaken every 10 min to keep them in suspension. Thereafter, cells were seeded in a 6 cm Petri-dish and incubated at 37°C and 5% CO_2_ for the remaining infection time.

### Cross-Presentation Assay

Murine Cloudman S91 melanoma cells (ATCC CCL-53.1; MHC I haplotype H-2^d^) were used as MHC I-mismatched feeder cells. Feeder cells were trypsinized, counted using a Neubauer cell counting chamber and 2 × 10^6^ feeder cells were spun down at 319 xg. After centrifugation, cell pellets were resuspended in 200 μl of M2 medium (RPMI 1640 containing 10% heat inactivated FCS, 50 μM 2-mercaptoethanol). Cells were infected at MOI 1 and incubated for 16 h at 37°C and 5% CO_2_. In the first hour of infection, feeder cells were softly shaken every 10 min to keep them in suspension and in the second hour of infection, every 20 min. Thereafter, cells were seeded in a 6-well plate and incubated at 37°C and 5% CO_2_ for the remaining infection time. Infected feeder cells and mock controls were treated with 0.3 μg/ml psoralen for 15 min prior to irradiation with UVA (PUVA) for 15 min at room temperature. 2 × 10^6^ feeder cells were washed and cocultured with 2 × 10^6^ BMDCs in a 6 cm Petri-dish at 37°C and 5% CO_2_. Cross-presenting BMDCs were analyzed at 12 h post co-cultivation for their ability to reactivate antigen-specific CD8^+^ T cell lines, described below, or at 20 h post co-cultivation for their SIINFEKL/K^b^-loading ability and maturation phenotype.

### CD8^+^ T Cell Activation Assay

Peptide-specific CD8^+^ T cell lines were used as read-out for the antigen presentation capacity of infected or non-infected cross-presenting BMDCs. 2 × 10^5^ CD8^+^ were co-cultured with 4 x 10^5^ BMDCs for 4 h in the presence of 1 μg/ml brefeldin A (Merck) at 37°C and 5% CO_2_. T cell activation of CD8^+^ T cells was determined by cytokine production analyzed by intracellular cytokine staining (ICS) as described below. For detection of SIINFEKL/K^b^ complexes at the cell surface, anti-SIINFEKL/K^b^ APC antibody (eBioscience 25-D1.16) was used after CD16/32-Fc-blockade (2.4G2, BD) and viability dye (Invitrogen). FACS analysis was performed on BD FACS CantoII and FlowJo 6.4.2 software.

### Generation and Maintenance of CD8^+^ T Cell Lines

LPS blasts were produced by incubating 1 × 10^6^ splenocytes/ml derived from naïve C57BL/6 mice with 25 μg/ml LPS (Sigma-Aldrich) and 7 μg/ml dextran-SO_4_ (Sigma-Aldrich) for 4 days at 37°C and 5% CO_2_. After irradiation of LPS-blasts with 30 Gy, cells were washed with RPMI 1640 medium and incubated in 1 ml RPMI 1640 medium containing 5 μg/ml β-microglobulin and 1 ng/ml of the appropriate peptide for 30 min at 37°C and 5% CO_2_. Peptides were A19_47−56_ (VSLDYINTM), B8_20−27_ (TSYKFESV), K3_6−15_ (YSLPNAGDVI), A3_270−277_ (KSYNYMLL), or D13_118−126_ (NCINNTIAL) derived from MVA and OVA_257−264_ (SIINFEKL) peptide derived from ovalbumin. K3 and D13-derived peptides are H2-D^b^-restricted, all other peptides are H2-K^b^- restricted. All peptides were purchased from Biosynthan (Germany). Cells were washed with medium prior to co-cultivation of 3 × 10^6^ peptide-loaded LPS-blasts with 7 × 10^6^ splenocytes from MVA-PK1L-OVA vaccinated C57BL/6 mice for 8 days at 37°C and 5% CO_2_.

CD8^+^ T cells were maintained by weekly restimulation using peptide loaded EL.4 cells (ATCC TIB-39), naïve splenocytes and M2 medium containing 5% TCGF [conditioned medium containing supernatant from rat splenocytes stimulated with 5 μg/ml Concanavalin A, produced as described (52)]. EL.4 cells and splenocytes were irradiated with 100 Gy or 30 Gy, respectively prior to peptide-loading and/or incubation with CD8^+^ T cells.

### Antibodies and Flow Cytometry

Cells were washed with PBS (Life Technologies, Darmstadt, Germany) and dead cells were excluded by viability dye staining (eBioscience^TM^ Fixable Viability Dye eFluor^TM^ 506) for 20 min on ice. Prior to the surface staining of CD8^+^ T cells or BMDCs, cells were washed twice with staining buffer (1% BSA, 0,02% NaN_3_ in PBS). To analyze activated CD8^+^ T cells, cells were stained for CD8 using anti-CD8-PB (eBioscience) for 30 min on ice. Thereafter, intracellular staining was performed, as described below. Alternatively, BMDCs were analyzed for their SIINFEKL/K^b^-loading ability using anti-CD11c-PE, -I-A/I-E-PB, -Kb-PE/Cy7, and -SIINFEKL/H2-K^b^-PE/Cy7 (all from eBioscience) or for their maturation phenotype using anti-CD11c-APC/Cy7, -CD86-APC, I-A/I-E-PE antibodies (all BD Pharmingen), or anti-CD40-PB antibody (Biolegend). Antibodies were added after blocking of Fc-receptors using CD16/32 antibodies (Fc-Block^TM^, BD Biosciences). After incubation on ice for 30 min, cells were washed and fixed with 1% paraformaldehyde and stored at 8°C until flow cytometry was performed using BD FACS Canto II (BD Biosciences, Heidelberg, Germany).

### Intracellular Staining (ICS)

Cells were washed twice and permeabilized for 15 min on ice using BD Cytofix/Cytoperm^TM^ solution. After an additional washing step, cells were incubated with anti-IFNγ and/or anti-MIP1-α (both Bioscience) for 30 min in the dark on ice. Finally, cells were washed and fixed with 1% paraformaldehyde. Flow cytometry was performed using BD FACS Canto II (BD Biosciences, Heidelberg, Germany).

### Phenotypical Analysis of Memory T Cell Subsets *ex vivo*

STING KO mice and wild-type littermates were immunized as described above. Spleens were removed 7 days after primary immunization. Isolated splenocytes were directly used for staining with B8- or OVA-reactive tetramers. Briefly, splenocytes were washed with PBS and dead cells excluded by viability dye staining (eBioscience^TM^ Fixable Viability Dye eFluor^TM^ 506). Thereafter, cells were washed twice with staining buffer (1% BSA, 0.02% NaN_3_ in PBS) and Fc-receptors blocked using CD16/32 antibodies (Fc-Block^TM^, BD Biosciences). After washing, cells were incubated with B8- and OVA-reactive tetramers for 15 min and incubated in the dark on ice. Anti-CD8-PB, -CD127-APC, and -CD62L-PE/Cy7 (all eBioscience) antibodies were added to the tetramer-stained cells and incubated for additional 20 min in the dark on ice. Samples were analyzed by flow cytometry using BD FACS Canto II (BD Biosciences, Heidelberg, Germany).

### Cytokine/Chemokine Response in Supernatants

Supernatants of MVA- or mock-infected BMDCs were harvested 12 h post-infection and supernatants of cross-presenting BMDCs were collected after overnight co-cultivation (12-15 h). Detection of IFN-α (LumiKine^TM^ mIFN-α) and -β (LumiKine^TM^ Xpress mIFN-β) was performed according to the manufacturer's protocol. LEGENDPlex^TM^ assays (Mouse Proinflammatory Chemokine Panel and Mouse Anti-Virus Response Panel Mix and Match Subpanel; Biolegend) were performed to monitor cytokine/chemokine expression. Briefly, spleens were collected and carefully homogenized in 500 μl ice-cold PBS plus protease inhibitor cocktail (Roche). The supernatants were collected after centrifugation and stored at −80°C till usage. A volume of 25 μl supernatant was used following the manufacturer's protocol.

For the analysis of cytokines and chemokines produced by MVA- or mock-infected feeder cells, supernatants were harvested at 12 h p. i. and analyzed according to the manufacturer's protocol. Samples were analyzed by flow cytometry on a FACS CantoII (BD Bioscience). Data were analyed using LEGENDPlex software (Biolegend).

### Generation of STING KO Feeder Cells

STING KO feeder cells were generated using CRISPR/Cas9 KO (TMEM173 CRISPR/Cas9 KO plasmid sc-428364) and HDR (TMEM173 HDR plasmid sc-428364-HDR) plasmids from Santa Cruz. Cells were seeded the day before in a 6-well plate and transfected at 80% confluency with both CRISPR/Cas9 plasmids using FuGENE HD Transfection Reagent according to the manufacturer's protocol. After 2 days cells were selected for 5 days with media containing 0.5 μg/ml puromycin (Sigma) which was renewed after 2 days of puromycin treatment. The phenotype of gene-edited cells was verified by western blot analysis, as described below.

### Phagocytosis Activity and Endosomal Function

4 × 10^6^ STING KO or WT BMDCs were transferred to a 6-well each and incubated with 10 μL of OVA-AF594 or OVA-DQTM (10 ng/mL) for 2 h at 37°C and 4°C. DQ-Ovalbumin is a self-quenching conjugate which shows a bright green fluorescence during proteolytic degradation. Cells were washed and stained with a vitality dye (eBioscience^TM^ Fixable Viability Dye eFluor^TM^ 660) as well as anti-CD11c-PE/Cy7 (eBioscience) and analyzed by flow cytometry using BD FACS Canto II (BD Biosciences, Heidelberg, Germany).

### Western Blot Analysis

The expression of STING protein was analyzed in order to verify gene-editing of TEMEM173 by CRISPR/Cas9. Cell lysates were prepared from STING KO and control feeder cells and a SDS-PAGE and blotting on nitrocellulose membranes performed, as described elsewhere (53). Membranes were blocked using 5% BSA in Tris-buffered saline containing 0.1% Tween 20 (Merck) for 1 h at room temperature. Rabbit anti-STING (cell signaling) and mouse anti-β-actin (Sigma) were diluted according to the manufacturer's protocol and incubated over night with the membrane. Incubation of peroxidase-conjugated goat anti-rabbit or anti-mouse IgG (Jackson) for 1 h at room temperature allowed the detection by chemoluminescence using Super Signal West Dura Chemoluminescent Substrate (Thermo Scientific).

### Statistical Analysis

All statistical analyses were performed using Graphpad Prism software version 7. Statistical comparisons of two groups were conducted using the unpaired, two-tailed Student's *t*-test. The results were depicted as mean ± standard deviation (SD) either pooled or representative from an indicated number of independent experiments. Statistical significance (P) is represented as: ^*^P ≤ 0.05; ^**^P ≤ 0.01; ^***^P ≤ 0.001; ^****^P ≤ 0.0001.

## Results

### STING Is Crucial for the Induction of Efficient CD8^+^ T Cell Responses After Infection With MVA

**Figure 1:**
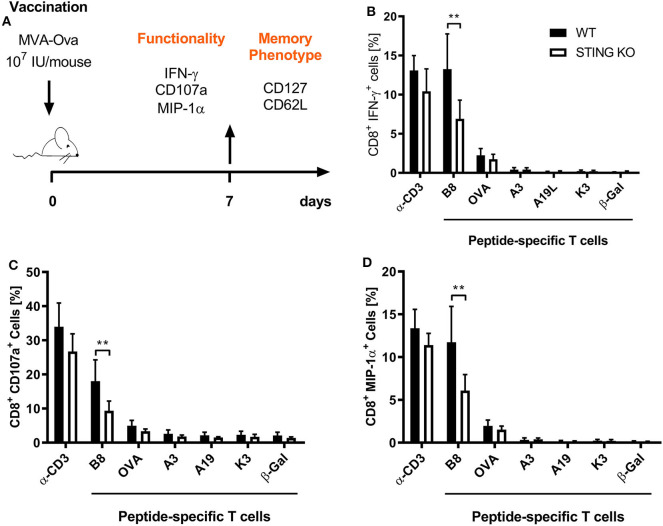
STING has a strong impact on shaping the immunodominance of MVA-specific primary immune responses particularly acting on the immunodominant B8 antigen. **(A)** STING KO mice (STING KO) and WT-littermates (WT) were vaccinated on day 0 i. p. with 10^7^ IU MVA-P7.5-OVA. Seven days after priming antigen-specific CD8^+^ T cells were analyzed *ex vivo* by flow cytometry for their functionality by expression of **(B)** IFN-γ, **(C)** CD107a, and **(D)** MIP-1α. Anti-CD3 antibody (α-CD3) and β-galactosidase (β-Gal) peptides were used for stimulation as positive and negative controls, respectively. Data are represented as mean ± SD of *n* = 8 mice per group pooled from three independent experiments. Statistical significance (*P*); **P ≤ 0.01.

**Figure 2:**
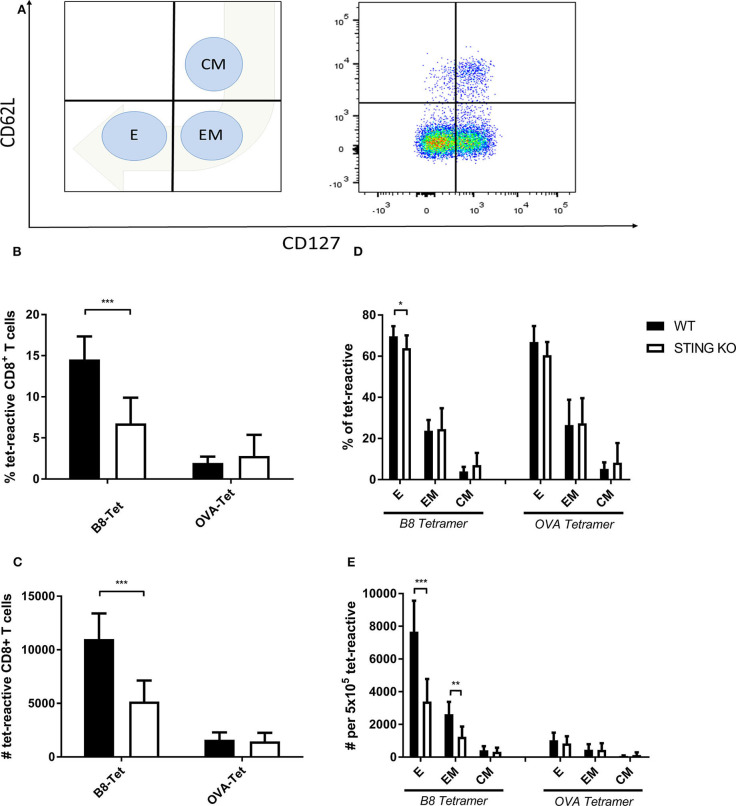
Maturation and differentiation of B8-specific immunodominant CD8^+^ T cells is STING-dependent. STING KO mice (STING KO) and WT-littermates (WT) were vaccinated on day 0 i. p. with 107 IU MVA-P7.5-OVA. Seven days after the prime antigen-specific CD8^+^ T cells were analyzed *ex vivo* by flow cytometry for their memory phenotype by expression of CD62L and CD127. **(A)** Presentation of the gating strategy and division into the individual memory subtypes. Analysis of the **(B)** relative frequency (%) and **(C)** absolute numbers of B8- or OVA-specific CD8^+^ T cell responses determined by tetramer staining (tet-reactive). In addition, the respective memory T cell subpopulations [effector T cells (E), effector memory T cells (EM) and central memory T cells (CM)] within the tetramer-reactive fraction are shown in **(D)** frequencies and **(E)** absolute numbers. B8 tetramer: *n* = 13; OVA tetramer: *n* = 11. Data are represented as mean ± SD of *n* = 13 (for B8 tetramer) or *n* = 11 (for OVA tetramer) mice per group pooled from three independent experiments for B. Statistical significance (P); *P ≤ 0.05; **P ≤ 0.01; ***P ≤ 0.001.

STING plays an important immunoregulatory role after recognition of cytosolic foreign DNA which is delivered by many DNA viruses including MVA. Next to innate immune activation, there may be STING-dependent effects on adaptive immunity such as CTL responses. In order to investigate a possible role of STING in MVA vaccination, we immunized STING KO and STING WT mice, which were genotypically confirmed by PCR (Supplementary Figure 1A), intraperitoneally (i.p.) or intramuscularly (i. m.) with 10^7^ IU ovalbumin (OVA)-expressing MVA (MVA-P7.5-OVA). On day 7, spleens were harvested, splenocytes shortly restimulated with peptides and subsequently used for intracellular cytokine staining and analyzed by flow cytometry (Figure 1A). CD8^+^ T-cells were examined for IFNγ, MIP-1α, and CD107a expression, respectively (Figures 1B–D). In addition, antigen-specific CD8^+^ T cells were determined for the recombinant antigen OVA and for vaccinia virus for the immunodominant viral antigen B8 by specific multimers and further discriminated into memory cell subpopulations (Figure 2A). Interestingly, the specific CD8^+^ T cell response against the immunodominant antigen B8 was significantly reduced in STING KO mice for all markers examined, while responses against subdominant viral epitopes (A3, K3, A19) as well as against OVA were comparable, yet with a slight tendency for a decrease in STING KO mice (Figures 1B–D). Moreover, we show that the dependency on STING as well as immunodominance of B8 was independent of the immunization route since primary B8-specific CD8^+^ T cell responses in STING KO mice were significantly decreased for all markers tested with the exception of MIP-1α after i.m. vaccination (Supplementary Figure 2). Interestingly, we additionally found decreased T cell frequencies for subdominant OVA and A3 epitopes with the ability for IL2 (OVA) or CD107a (OVA and A3) expression which we had not seen before after i.p. administration. In addition to the functional analysis, antigen-specific memory cell subpopulations were phenotypically analyzed during the acute phase. Differentiation between central and effector memory cells as well as effector T cells was carried out by monitoring the activation and maturation markers CD62L (L-selectin) and CD127 (α subunit of the Interleukin-7 receptor) within the multimer-positive (antigen-specific) CD8^+^ T cell population (Figure 2A). The overall frequency (Figure 2B) as well as absolute numbers (Figure 2C) of B8-specific CD8^+^ T cells in STING KO mice, as determined by B8-specific tetramer reactivity, were significantly reduced compared to STING WT mice. Interestingly, predominantly short-lived effector T cells were reduced in frequency (Figure 2D) and absolute numbers (Figure 2E). Furthermore, B8-specific effector memory CD8^+^ T cells were significantly reduced in absolute numbers (Figure 2E). In contrast, frequencies and distribution of memory subpopulations were comparable for OVA tetramer-reactive CTL in STING KO or WT mice.

**Figure 3:**
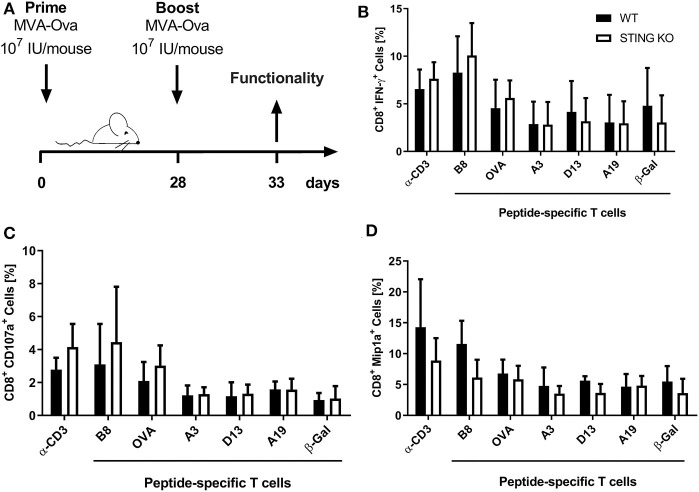
STING is not relevant for developing long term memory and efficient secondary expansion of CD8^+^ T cell responses in prime/boost vaccination. **(A)** STING KO mice (STING KO) and WT-littermates (WT) were vaccinated i. p. with 10^7^ IU MVA-P7.5-OVA on day 0 (prime) and 28 (boost). Five days after the boost antigen-specific CD8^+^ T cells were analyzed *ex vivo* by flow cytometry for their functionality by expression of **(B)** IFN-γ, **(C)** CD107a, and **(D)** MIP-1α. Anti-CD3 antibody (α-CD3) and β-galactosidase (β-Gal) peptides were used for stimulation as positive and negative controls, respectively. Data are represented as mean ± SD of *n* = 9 mice per group pooled from three independent experiments.

Moreover, STING KO mice and WT-littermates were vaccinated i. p. with 10^7^ IU MVA-P7.5-OVA on day 0 (prime) followed by a secondary immunization at day 28 (boost). Five days after the boost antigen-specific CD8^+^ T cells were analyzed *ex vivo* by flow cytometry for their functionality (Figure 3A). As expected, since we failed to see an influence of STING for developing long-term memory, STING was neglectable for efficient secondary expansion as indicated by comparable functionality of CD8^+^ T cell responses after MVA prime/boost vaccination in KO or WT mice (Figures 3B–D).

### Effector T Cell Activation by STING KO BMDCs Is Not Impaired *in vitro*

**Figure 4:**
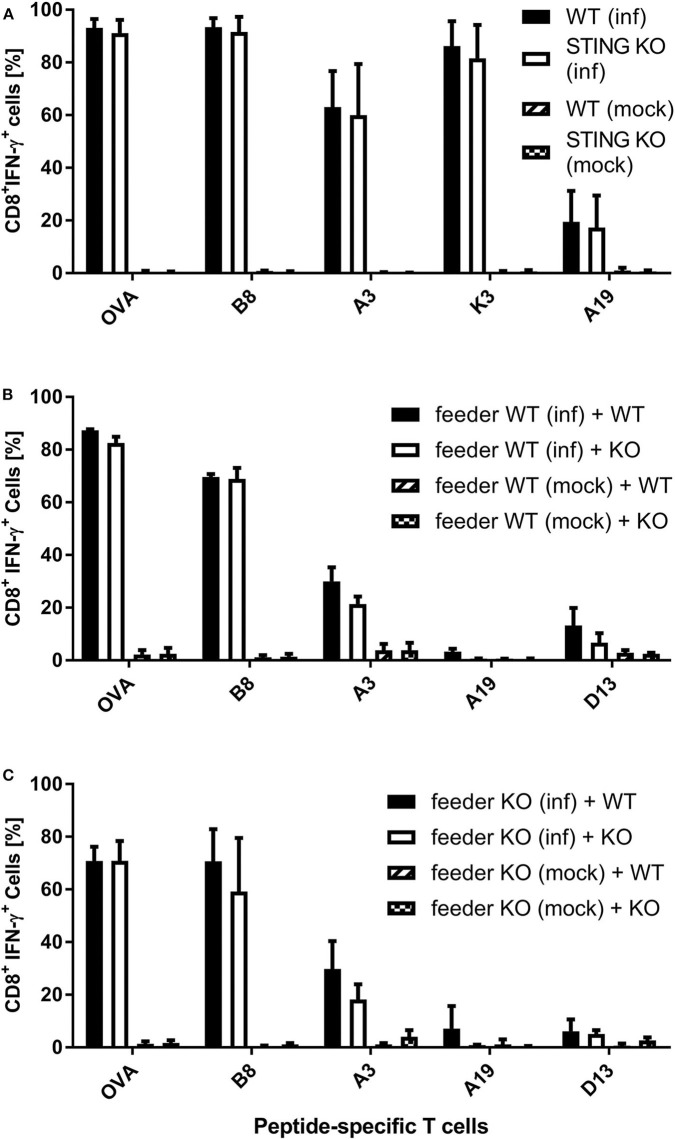
Re-activation of antigen-specific CD8^+^ T cells by infected or cross-presenting STING KO BMDC is not impaired. **(A)** BMDCs from STING KO mice (STING KO) or wildtype-littermates (WT) were infected with MVA-PK1L-OVA at MOI 1 (inf) or mock infected (mock) for 12 h. **(B)** MHC I-mismatched wildtype feeder cells (feeder WT) expressing STING were infected with MVA-PK1L-OVA at MOI 1 (inf) or mock infected (mock) for 12 h, treated with PUVA, washed and co-cultured with BMDCs derived from either STING KO mice (KO) or WT littermates (WT). **(C)** MHC I-mismatched STING KO (feeder KO) were infected with MVA-PK1L-OVA at MOI 1 (inf) or mock infected (mock), treated with PUVA, washed and co-cultured with BMDCs generated from either STING KO (KO) or wildtype mice expressing STING (WT). **(A)** BMDCs were analyzed 12 h post-infection or **(B,C)** 12 h post co-cultivation for their ability to activate CD8^+^ T cell lines to produce IFN-γ as determined by ICS followed by FACS analysis. CD8^+^ T cell activation was determined as frequency of IFN-γ expressing T cells specific for the indicated peptides derived from OVA or MVA antigens (B8, A3, K3, A19, D13). Data are represented as mean ± SD of **(A)** *n* = 3 or **(B,C)** *n* = 2 BMDC preparations from individual mice per group pooled from **(A)** three or **(B,C)** two independent experiments.

It has been shown recently that both direct as well as cross-presentation of antigen is important for the induction of optimal CD8^+^ T cell responses during MVA vaccination (42). To elucidate the underlying mechanisms of the impaired CD8^+^ T cell response we had observed in STING KO mice during infection with recombinant MVA, we first analyzed the ability of STING KO and WT bone-marrow-derived dendritic cells (BMDCs) to reactivate CD8^+^ T cells *in vitro* in the setting of antigen cross- as well as direct presentation (Supplementary Figures 1B, 3). The purity of BMDC cultures and the expression of maturation markers prior to experimental use was comparable for STING KO or WT (Supplementary Figures 4A,B). By using CD8^+^ T cell lines, specific for the viral antigens B8, K3, A3, and A19 or the recombinant antigen OVA, we observed a comparable T cell activation by infected STING KO and WT BMDCs (direct presentation) (Figure 4A). In order to investigate the antigen presentation capability of cross-presenting BMDCs, we used MVA-infected MHC I-mismatched feeder cells unable to directly present antigen to CTL lines. BMDC derived from WT (Supplementary Figure 4C) and KO mice (Supplementary Figure 4D) showed comparable marker expression profiles in the cross-presentation setting when co-cultivated with mock infected feeder cells. Only cocultivation with infected feeder cells changed the BMDC phenotype. Washing and PUVA treatment of infected feeder cells prevented false-positive results caused by directly antigen presenting BMDCs accidentally infected with input virus. After co-culturing STING KO or WT BMDCs with MVA-infected feeder cells, we did not observe any impact of STING deficiency in cross-presenting BMDCs on T cell activation for all epitope-specificities tested (Figure 4B). Considering the possibility that the phenotype of feeder cells may have an impact on their ability to be cross-presented e.g., by licensing DCs for cross-presentation, we generated STING KO feeder cells (Supplementary Figure 5) and tested these cells as an antigenic source for BMDCs for cross-presentation. Interestingly, all CD8^+^ T cell-specificities showed comparable IFN-γ production after incubation with WT BMDC co-cultured with either STING KO or WT feeder cells (Figure 4C). Since our T cell lines represent pre-activated effector cells known to become already activated by a low epitope density on the cell surface of antigen-presenting cells, it was likely that there may be differences in the antigen presentation ability of STING-deficient BMDCs which were not obvious in the cytokine production assay (ICS) such as the expression rate or density of peptide/MHC I-complexes at the cell surface.

### STING Deficiency Results in a Reduced Antigen Processing and Presentation Capacity

**Figure 5:**
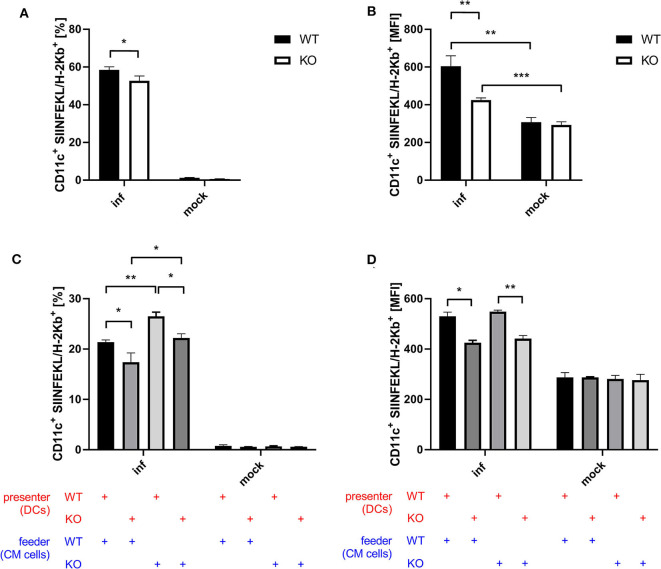
STING in BMDC is crucial for efficient antigen processing and presentation while STING in feeder cells increases the size of the DC pool available for cross-presentation. **(A,B)** GM-CSF-BMDCs generated from STING KO mice (KO) or WT-littermates (WT) were infected with MVA-PK1L-OVA at MOI 1 (inf) or mock infected (mock). At 12 h post-infection SIINFEKL/K^b^ surface expression was determined. **(C,D)** The SIINFEKL/K^b^ loading ability of cross-presenting BMDCs was analyzed after co-culturing MVA-infected STING KO (feeder KO) or WT Cloudman (CM) feeder cells (feeder WT) with either STING KO (presenter KO) or WT BMDCs (presenter WT) for 20 h. Data are representative for one of two independent experiments and are presented as **(A,C)** frequencies (%) or **(B,D)** mean fluorescent intensities (MFI) and represent the mean ± SD of *n* = 3 BMDC preparations from individual mice per group. Statistical significance (*P*); *P ≤ 0.05; **P ≤ 0.01; ***P ≤ 0.001.

Next to CD8^+^ T cell activation as a more indirect effect of antigen-presentation by BMDCs, we examined antigen processing and presentation by monitoring the surface expression of SIINFEKL peptide/K^b^ complexes of BMDCs after infection with recombinant MVA expressing OVA. Infected STING KO BMDCs showed a significantly reduced frequency of SIINFEKL/K^b^ positive cells (Figure 5A) as well as a decreased density of SIINFEKL/K^b^ complexes as compared to STING WT BMDCs (Figure 5B). To study whether antigen processing and presentation is altered in cross-presenting STING deficient BMDCs, we performed an assay with a setting similar to the cross-presentation assay described above using either MVA-infected STING KO or WT feeder cells which were co-cultured either with non-infected STING KO or WT BMDCs, but without adding CTL. Interestingly, we observed significant differences in the ability to produce SIINFEKL/K^b^ complexes for both settings with STING deficiency on either presenter BMDC or feeder cell side (Figures 5C,D). The lack of STING in BMDCs only resulted in significant decrease of SIINFEKL/K^b^ expressing cells (Figure 5C) as well as significant reduction of the amount of SIINFEKL/K^b^ complexes on individual STING KO BMDCs compared to WT BMDCs (Figure 5D). Unexpectedly, co-incubation of infected STING KO feeder cells resulted in a highly significant increase in SIINFEKL/K^b^ expressing BMDCs which was higher in STING WT compared to KO cells (Figure 5C). Of note, increased MFIs for SIINFEKL/K^b^ were mainly depending on STING in BMDCs (Figure 5D) indicating that the presence or absence of STING in feeder cells had only a limited impact on the amount of SIINFEKL/K^b^ complexes at the cell surface of BMDCs. Nevertheless, there was a significant reduction, when BMDCs were STING-deficient (Figure 5D). Taken together, these data indicate that STING has a strong impact on the efficiency of antigen processing and presentation in directly infected as well as cross-presenting BMDCs. In addition, the absence of STING in infected feeder cells, serving as a source for antigen, had no impact on the efficiency of antigen presentation, but significantly enhanced the number of BMDCs which were able to cross-present antigen. This may be an indication that STING is highly relevant for licensing feeder cells to activate DCs for cross-presentation, while STING in cross-presenting DCs supports their efficiency in SIINFEKL/K^b^ loading and/or presentation.

**Figure 6:**
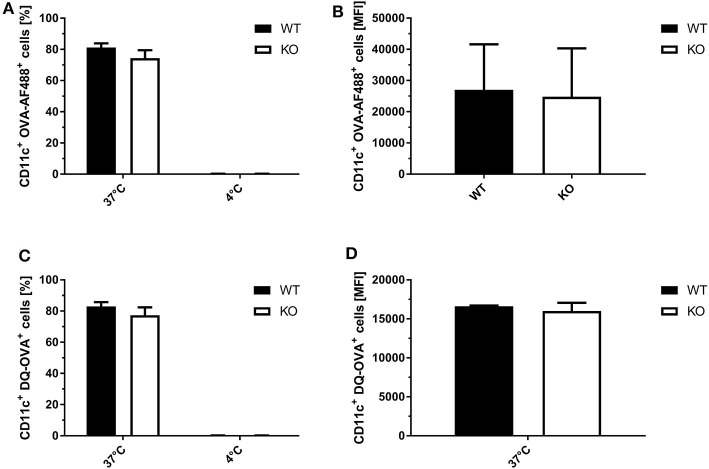
STING KO and WT BMDCs showed comparable phagocytosis activity and endosomal function. **(A)** GM-CSF-BMDCs were generated from STING KO (KO) mice or wildtype littermates (WT). The phagocytic activity was analyzed using **(A,C)** OVA-AF488 or **(B,D)** DQ-OVA at 4 and 37°C. In addition, DQ-OVA **(B,D)** allowed to measure proteolytic activity resulting in increased fluorescence. Data are presented as **(A,B)** frequencies (%) or **(C,D)** mean fluorescent intensities (MFI) and represent the mean ± SD of *n* = 3 BMDC preparations from individual mice per group pooled from three independent experiments.

To exclude that the observed differences in SIINFEKL/Kb surface expression in STING KO and WT BMDCs were not due to differential phagocytosis activity or endosomal function, we analyzed the ability of STING-deficient and WT BMDCs to endocytose and process fluorophore-conjugated OVA. Using OVA labeled with either the fluorophore AF488 or DQ, we observed comparable numbers of OVA-AF488^+^ CD11c^+^ (Figure 6A) or DQ-OVA^+^ CD11c^+^ cells (Figure 6B) for STING KO and control BMDC cell cultures at 37°C. In addition, STING KO and WT BMDCs endocytosed comparable amounts of OVA-AF488^+^ (Figure 6C) as well as DQ-OVA (Figure 6D, Supplementary Figure 6), indicating a similar phagocytic and endosomal activity.

### STING Supports Efficient DC Maturation

**Figure 7:**
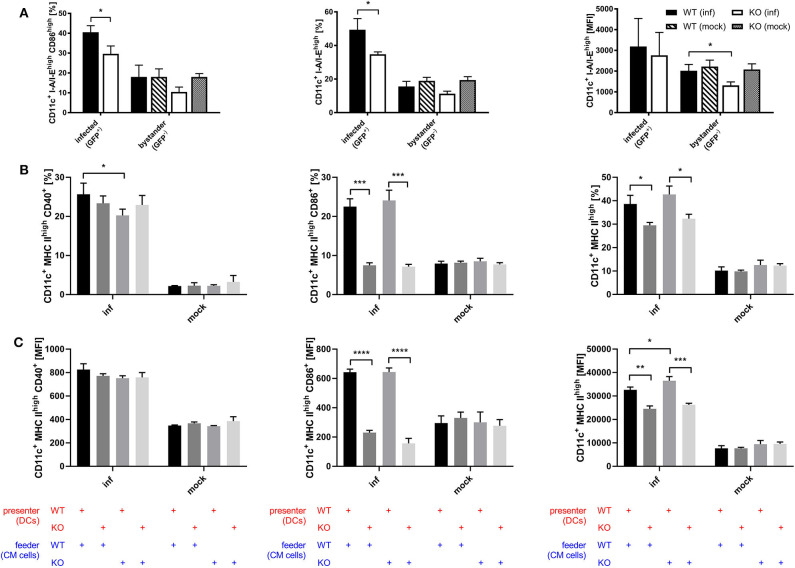
STING strongly supports DC maturation. **(A)** GM-CSF-BMDCs were generated from STING KO mice (KO) and WT-littermates (WT), directly infected with MVA-P7.5-NP-SIINFEKL-eGFP at MOI 5 and analyzed 12 h post-infection for the expression of CD86 and MHC II. Here, infected BMDC were determined by GFP expression (GFP+), whereas accompanying non-infected bystander DC showed no GFP expression (GFP-). **(B,C)** The maturation of cross-presenting BMDCs was analyzed after co-culturing of MVA-PK1L-OVA-infected STING KO (feeder KO) or WT feeder cells (feeder WT) at MOI 1 with either STING KO (presenter KO) or WT BMDCs (presenter WT) for 20 h. Data are depicted as **(B)** frequencies (%) or **(C)** mean fluorescent intensities (MFI) for the expression of CD40, CD86, and MHC II and are presented as mean ± SD of *n* = 3 BMDC preparations from individual mice per group **(A)** pooled from three independent experiments or **(B,C)** representative for one of two independent experiments, each with *n* = 3 BMDC preparations from individual mice per group. Statistical significance (*P*); *P ≤ 0.05; **P ≤ 0.01; ***P ≤ 0.001; ****P ≤ 0.0001.

Considering the connection between type I interferon and maturation of DCs which has been shown by previous research (54–57), we assessed whether STING is involved in the maturation process of DCs during infection with recombinant MVA by analyzing the BMDC maturation phenotype in the direct infection as well as the cross-presentation setting. Using recombinant MVA expressing SIINFEKL linked to eGFP for the infection of STING KO and WT BMDCs, we were able to distinguish infected (GFP-positive) from uninfected bystander BMDCs (GFP-negative). Interestingly, we observed that STING deficiency resulted in significantly reduced CD86 and MHC II expression in infected BMDCs and a significant decrease in the amount of MHC II (MFI) in MHC II high expressing BMDCs in the bystander population (Figure 7A). These results indicate that signaling via STING is not only relevant for maturation of directly infected BMDCs, but is also important for the maturation of bystander BMDCs. Furthermore, we tested if the presence or absence of STING in feeder cells or cross-presenting BMDCs has an effect on the maturation of BMDCs by co-culturing either MVA-infected STING KO or WT feeder cells with non-infected STING KO or WT BMDCs in a cross-presentation assay-like setting but without CTL (Figures 7B,C). CD40-expressing mature BMDCs were significantly diminished when infected STING-deficient feeder cells were used for co-cultivation, while there was no effect on CD86 or MHC II expression, if STING was absent in feeder cells (Figure 7B). STING deficiency in BMDC did not affect CD40 expression (Figure 7B), nor CD40 expression levels (MFI) of all groups (Figure 7C). In contrast, when STING was absent in BMDCs, we did not only observe a massive drop in CD86 and MHC II high expressing cells (Figure 7B), but also found a significantly reduced amount of these markers (MFI) on STING KO BMDCs (Figure 7C). Our data suggest that STING supports efficient maturation of directly infected BMDCs as well as bystander and cross-presenting BMDCs during infection with recombinant MVA.

### STING Is Crucial for the Induction of Type I Interferon *in vitro* and *in vivo*

**Figure 8:**
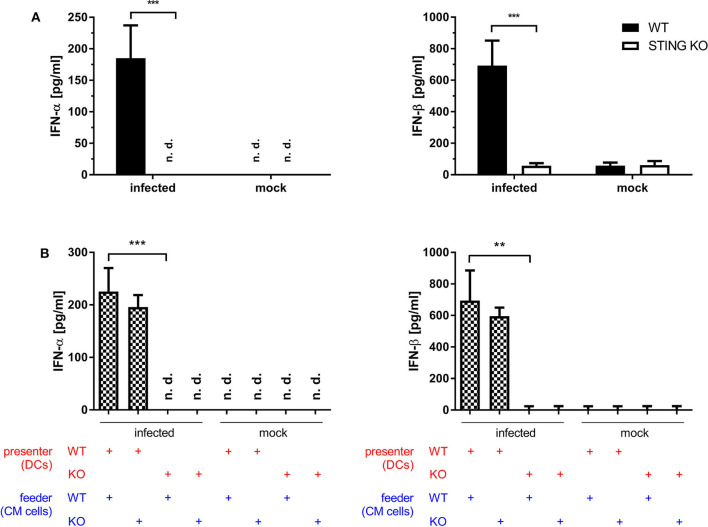
STING is crucial for the induction of type I interferon *in vitro*. **(A)** GM-CSF-BMDCs generated from STING KO mice or WT-littermates were either directly infected with MVA-PK1L-OVA at MOI 1 or mock infected or **(B)** left uninfected, but co-cultivated with feeder cells which were infected with MVA-PK1L-OVA at MOI 1 for 12 h or left uninfected (mock). Supernatants were collected at **(A)** 12 h post-infection or **(B)** 12 h post co-cultivation and **(A,B)** concentrations of IFN-α and -β were determined by ELISA. In the co-culture setting, supernatants from co-cultures with either infected STING KO (feeder KO) or wildtype feeder cells (feeder WT) with either uninfected STING KO (presenter KO) or WT BMDCs (presenter WT) were taken at 12 h post co-culturing and tested. Data represent the mean ± SD of **(A)** *n* = 4 and **(B)** *n* = 3 BMDC preparations from individual mice per group pooled from three independent experiments. Statistical significance (*P*); **P ≤ 0.01; ***P ≤ 0.001; n. d. = not detected.

**Figure 9:**
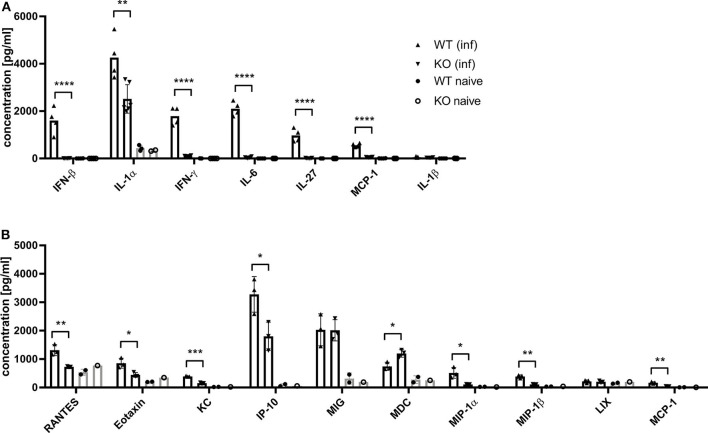
MVA-vaccinated STING KO mice show a decreased cytokine and chemokine response. C57BL/6 mice (WT) and STING KO (KO) were i. p. infected with 1 × 10^8^ IU MVA-PH5-OVA. After 6 h of infection, spleens were harvested, cell suspensions prepared and **(A)** cytokines or **(B)** chemokines were determined in spleen supernatants by cytoplex assay. Data from infected groups (WT inf, KO inf) are represented as mean ± SD of *n* = 4-6 (cytokines) and *n* = 3 (chemokines) mice per group pooled from two independent experiments. Naïve STING KO (KO naïve) and wildtype (WT naïve) mice were used as control and data are depicted as mean ± SD of *n* = 2-3 (cytokines) and *n* = 2 (chemokines) mice per group. Statistical significance (*P*); *P ≤ 0.05; **P ≤ 0.01; ***P ≤ 0.001; ****P ≤ 0.0001.

Dai et al. (37) demonstrated that the type I interferon response in BMDCs after infection with MVA is completely STING-dependent. Consistent with these results, we observed a completely abrogated interferon-α (IFN-α) and -β (IFN-β) response determined *in vitro* in supernatants from infected STING-deficient BMDCs (Figure 8A). Similarly, cross-presenting BMDCs had no IFN-α and -β response when they were deficient for STING (Figure 8B). However, the presence or absence of STING in feeder cells had no impact on the induced type I interferon response in cross-presenting BMDCs (Figure 8B). Importantly, the observed type I interferon response arose from BMDCs only, because feeder cells were unable to produce type I interferons with or without MVA infection (Supplementary Figure 7). In line with the above findings, we observed the complete loss of IFN-β production when using spleen supernatants from vaccinated STING KO mice (Figure 9A). Furthermore, we saw a significantly reduced cytokine (Figure 9A) as well as chemokine response in STING-deficient mice (Figure 9B) affecting proinflammatory cytokines such as IL-1α, IL-6, MCP-1 (CCL2), IL-27 as well as IFN-γ (Figure 9A) and chemokines involved in T cell recruitment such as RANTES, MIP-1α/β, and MDC (Figure 9B).

## Discussion

The induction of a potent long-lasting antigen-specific CD8^+^ T cell response is a fundamental aim of prophylactic and therapeutic vaccination strategies against many intracellular pathogens such as viruses. To achieve this goal, it is essential to understand the mechanisms and factors which determine the quality and quantity of vaccine-induced CD8^+^ T cell immunity. Dai et al. (37) recently demonstrated that the production of type I interferon after infection of BMDCs with MVA *in vitro* was strictly STING-dependent. We were interested whether STING is also involved in the generation of an efficient type I interferon response *in vivo* and relevant for CD8^+^ T cell immunity after viral vector vaccination using recombinant MVA. We showed in this study that an intact STING signaling pathway was crucial for the priming and development of a fully functional vaccinia virus-specific CD8^+^ T cell response particularly in the acute phase (Figures 1, 2). The absence of STING in vaccinated mice led to an overall reduced CD8^+^ T cell response, but particularly affected the immunodominant B8-specific CTL response which is directed against an early gene product (Figure 1, Supplementary Figure 2). The entire genome replication of vaccinia virus and subsequent viral intermediate and late gene expression and translation takes place in organelles called viral factories (3, 58, 59). As a consequence late viral antigens such as A19 or D13 induce low CD8^+^ T cell responses, not only because of a delayed expression kinetic in infected cells, but also due to the detention of late antigens in viral factories (Figure 4A) (50). Likewise, late antigens showed a reduced availability for cross-presentation by antigen presenting cells during infection (Figures 4B,C) (50, 60, 61). On the other hand, our *in vivo* data correlate with the results obtained from the phenotypical analysis of BMDC maturation (Figure 7). Since cross-priming plays a decisive role for the quality and quantity of the CD8^+^ T cell response generated by MVA vaccination (42, 43), an intact and effective bystander BMDC maturation is likely essential. In fact, recent research demonstrated that the initial activation of CD4^+^ and CD8^+^ T cells is accomplished through both directly infected DCs and non-infected bystander DCs (42, 62). In this context, non-infected XCR1^+^ (CD8α^+^) DCs were critical and responsible for the initial activation of naive CD8^+^ T cells by cross-priming at later time points of infection and were supported by previously primed CD4^+^ T cells (42, 62). Notably, a study by Brewitz et al. (63) indicates that also pDCs play a key role for a potent CD8^+^ T cell response during viral infection. In particular, pDC depletion induced in Clec4c^+^/DTR mice significantly impaired the host immune response to MVA infection. Animals without pDCs showed significantly reduced B8-specific IFN-γ-producing CD8^+^ T cells which were mainly attributed to a lack of maturation of non-infected bystander (cross-presenting) XCR1^+^ DC population due to a reduction of pDC-derived IFN-α. Interestingly, this was a strictly local effect as the total amount of IFN-α in the lymph nodes remained unaltered (63). Furthermore, we demonstrate for i.p. and i.m administration that the reduced function of CD8^+^ T cells affected cytokine and chemokine production (Figure 1, Supplementary Figure 2). IFN-γ, CD107α and dependent on the route also MIP-1α were significantly reduced in immunodominant B8-specific CD8^+^ T cells derived from STING KO compared to WT mice suggesting a broader functional impairment. The establishment of immunodominance depends on various factors (e.g., naïve T cell precursor frequency, T cell avidity, antigen processing efficacy, epitope binding affinity, etc.) which most likely differ for each ligand. As recently shown (64), there may be variations between vaccinia virus vaccine delivery platforms such as replication-competent vs. -deficient viruses, different insertion loci for target gene expression or the presence/absence of viral thymidine kinase by which immunodominance can be shaped to a certain degree, but it will not be reversed even in the absence of the immunodominant B8 epitope (49). In contrast to the systemic i.p. route, peripheral i.m. application resulted in additional decrease of T cells specific for some subdominant epitopes such as OVA and A3 in STING KO mice. A possible explanation may be the lower amount of CD86 on STING KO BMDCs making costimulation a limiting factor in these mice. This hypothesis is supported by Lin et al. (65) demonstrating that immunodominance was sharpened by peripheral i.d. or s.c. application of replication-competent vaccinia virus strain WR, but immunodomination of B8 could be reduced using recombinant WR expressing CD80 and CD86 for priming.

A potent activation of CD8^+^ T cells leading to clonal expansion and effector function requires three signals: (I) TCR/antigen/MHC-I complex formation, (II) co-stimulatory receptor/ligand binding and (III) cytokine signals. STING-deficiency in infected as well as in cross-presenting BMDCs resulted in a decreased ability and efficiency in SIINFEKL/K^b^-loading (Figures 5A,B), indicating defects in antigen processing and presentation. Importantly, we demonstrated that phagocytosis and endocytic uptake and degradative processing was not impaired in BMDCs when STING was absent (Figure 6, Supplementary Figure 5). However, STING-deficiency in these APC could not impair activation of antigen-specific CD8^+^ T cell lines *in vitro* (Figure 4). Since effector CD8^+^ T cells including our T cell lines need rather low epitope densities on the cell surface of antigen-presenting cells to become reactivated (66, 67), even significant differences in the amount of peptide/MHC I-complexes presented by STING KO and control BMDCs will result in comparable reactivation of primed or memory T cells. In contrast, priming of naïve T cells will be much more sensitive to reduced amounts of peptide/MHC I complexes and/or co-stimulatory molecules (68) which is in line with the reduced primary T cell response seen *in vivo* after vaccination (Figures 1, 2). Similarly to infected BMDCs, the SIINFEKL/K^b^-loading ability and efficacy of cross-presenting BMDCs was only impaired when STING was absent in the BMDC (Figures 5C,D). In addition, we showed that the STING phenotype of feeder cells had no impact on the epitope density of cross-presenting BMDCs (Figure 5D). Surprisingly, we noticed that STING deficiency in feeder cells appeared to be highly beneficial for the activation of cross-presenting BMDCs promoting a dramatic increase of SIINFEKL/K^b+^ BMDCs (Figure 5C), suggesting that either alternative sensing pathways for MVA in STING KO feeder cells may be active (69) or STING in feeder cells may alter the ability of BMDCs to execute cross-presentation.

Optimal activation of T cell responses not only depends on the peptide/MHC complex density, but also on the correct DC phenotype namely mature or immature (63, 70). Maturation involves the expression of co-stimulatory molecules as well as the upregulation of MHC molecules, particularly MHC class II. Both are crucial for the generation of efficient T cell responses. CD86 which binds CD28 and CTLA-4 (71, 72) as well as CD40 interacting with CD40L (73) are important ligands for co-stimulation. We observed a dramatically reduced maturation in STING-deficient BMDCs with regard to the expression of CD86 and MHC II in directly as well as bystander and cross-presenting BMDCs, respectively (Figure 7). Interestingly, CD40 expression was not dependent on STING in BMDCs, but was significantly reduced when infected STING-deficient feeder cells were used in the cross-presentation setting indicating that exogenous STING-dependent signals other than type I IFN which was absent in feeder cells contributed to DC maturation.

Previous research has shown that many melanoma cells and cell lines have variable defects in interferon signaling pathways (74–76), which was also observed in the melanoma cell line that we used as feeder cells for this study (77, 78). Moreover, compared to infected BMDCs, we observed in MVA-infected S91 feeder cells a severely limited cytokine/chemokine response with a complete lack of type I and II interferons (Supplementary Figure 7). We anticipate that the most important factor related to STING deficiency in this model is likely the missing third signal from cytokines such as type I and type II interferons. In T cells, type I interferon triggers many important signaling cascades that are responsible for survival, proliferation and differentiation of effector cells (71, 79). An intact type I interferon response is essential for effective cross-priming *in vitro* and *in vivo* (80–82), and especially STING has been shown to be crucial for the expression of type I interferon (38, 39). In line with the findings of Dai et al. (37), we observed that production of type I interferon was completely STING-dependent in directly (Figure 8A) as well as cross-presenting BMDCs (Figure 8B). Ma et al. (83) showed that IFNAR1 knockout macrophages permit a lower induction of IFN-α4 and IFN-β compared to wildtype macrophages. They concluded that an intact IFNAR feedback loop was mandatory for optimal production of cGAMP-dependent type I interferons (83). It is also well-known that type I interferon suppresses the expression of various cytokines, including IL-12, and promotes the simultaneous expression of IFN-α and -β through a positive feedback loop (84, 85). In this respect, Frenz et al. (86) were able to demonstrate that MVA infection in IFNAR knockout mice triggers the induction of IL-12 but fails to induce a type I interferon response. We therefore conclude that type I interferon and possibly other cytokines induced by MVA in a STING-dependent manner may act simultaneously on CD8^+^ T cells as well as on dendritic cells to promote optimal T cell responses after a single vaccination *in vivo*.

In addition, STING was particularly important for the expansion/proliferation of immunodominant B8-specific CD8^+^ T cells (Figures 1B–D, 2B,C). Importantly, short-lived effector as well as effector memory T cells were significantly reduced in STING KO mice (Figures 2D,E). Berard et al. (87) and Mattei et al. (88) described that type I interferon together with IL-15 promote the homeostatic proliferation and maintenance of CD8^+^ T memory cells *in vivo*. Similarly, IFN-α promotes the development of human CD8^+^ T cells into memory cells (89). These findings correlate with our data indicating an impaired development of short-lived T cell memory with significant decrease of effector memory (EM) T cells (Figure 2E). This underlines the importance of STING-mediated type I interferon production during MVA infection for the quality of the acute response including effector and effector memory T cells. Interestingly, STING deficiency did not affect the long-term memory response mediated by central memory (CM) T cells (Figures 2D,E). The establishment of comparable long-term memory was corroborated by comparable T cell expansion seen after boost vaccinations at day 28 after priming in STING KO and WT mice (Figure 3), which indicates that STING is not required for efficient reactivation of memory T cells after secondary vaccination. It may be speculated that this also implies full protective capacity of vaccine-induced memory CTL against pathogen encounter.

Thus, STING signaling seems crucial for the innate and adaptive immune defense as demonstrated by the deficit production of a large array of cytokines and chemokines affecting the quality and quantity of relevant APC as well as anti-viral CD8^+^ T cells. STING plays a decisive role for the primary induction and development of CD8^+^ T cell responses in the acute phase during MVA infection *in vivo* which may allow for more rapid control and elimination of relevant pathogens in the vaccination setting. However, STING seems to be dispensable for the development of long-term CTL memory.

## Data Availability Statement

All datasets generated for this study are included in the article/Supplementary Material.

## Ethics Statement

The animal study was reviewed and approved by North Rhine-Westphalia State Agency for Nature, Environment and Consumer Protection (LANUV), Germany.

## Author's Note

Parts of the study have been presented at the 17th International Congress of Immunology, Beijing, China, and an abstract is available as a conference paper at https://onlinelibrary.wiley.com/doi/abs/10.1002/eji.201970400.

## Author Contributions

CB, GC, and ST conducted experiments and analyzed the data. RT generated recMVA. DB provided multimers. LJ provided STING KO mice and WT littermates. ID designed the study and interpreted the data. ID, CB, and GC wrote the manuscript. All authors contributed to the article and approved the submitted version.

## Conflict of Interest

The authors declare that the research was conducted in the absence of any commercial or financial relationships that could be construed as a potential conflict of interest.
