# Supplementary material for: Efficient Induction of Cytotoxic T Cells by Viral Vector Vaccination Requires STING-Dependent DC Functions
Source: Front Immunol. 2020 Jul 16;11:1458. doi: 10.3389/fimmu.2020.01458 (PMC7381110; doi:10.3389/fimmu.2020.01458)
Supplement: Supplementary file 1 [file Data_Sheet_1.PDF]

Supplementary Material

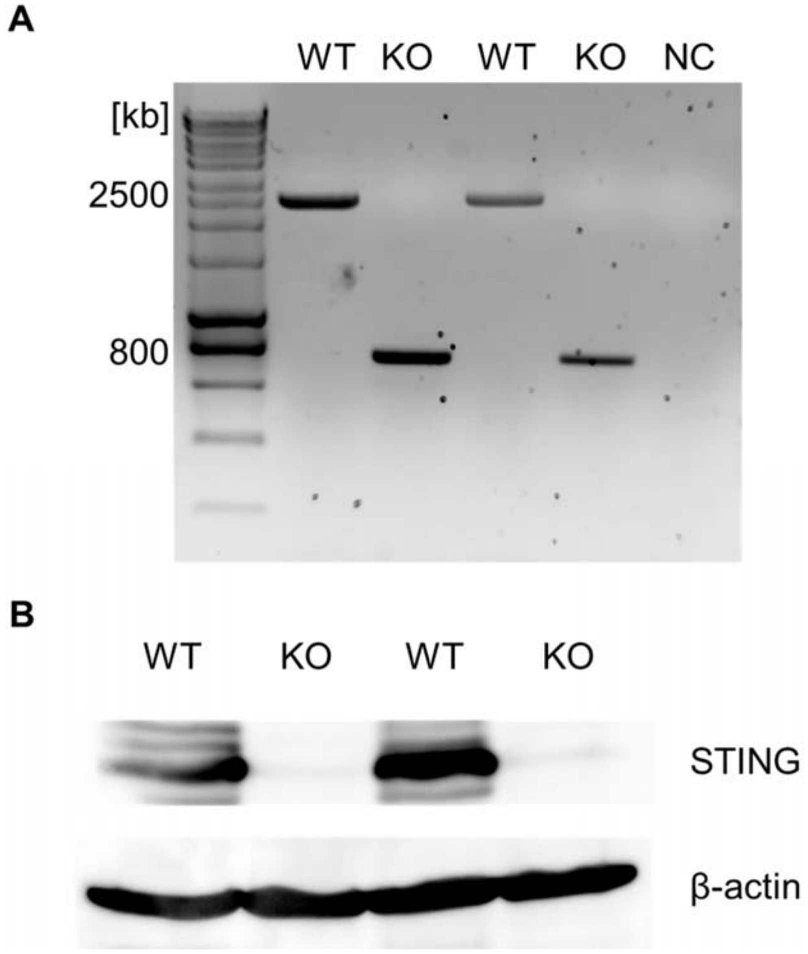

Supplementary Figure 1: Genotypic and phenotypic analysis of STING deficiency in STING KO mice and BMDC. (**A**) STING KO (KO) mice and WT littermates (WT) were genotyped using primers Sa-left (5'-ctggaccttttccccaaact-3'), neo (5'-gccttcttgacgagttcttc-3') and Sa-right (tttcatctgccttccaggtc-3'). Primers Sa-left and Sa-right amplified a 2.4 kb fragment in WT samples and primers neo and Sa-right resulted in a 800 bp fragment in KO samples. (**B**) GM-CSF-BMDCs were generated from STING KO (KO) mice or WT littermates (WT) and the STING-specific phenotype confirmed by Western blot analysis. KO BMDCs preparations showed deficient STING expression in comparison to control BMDCs (WT).

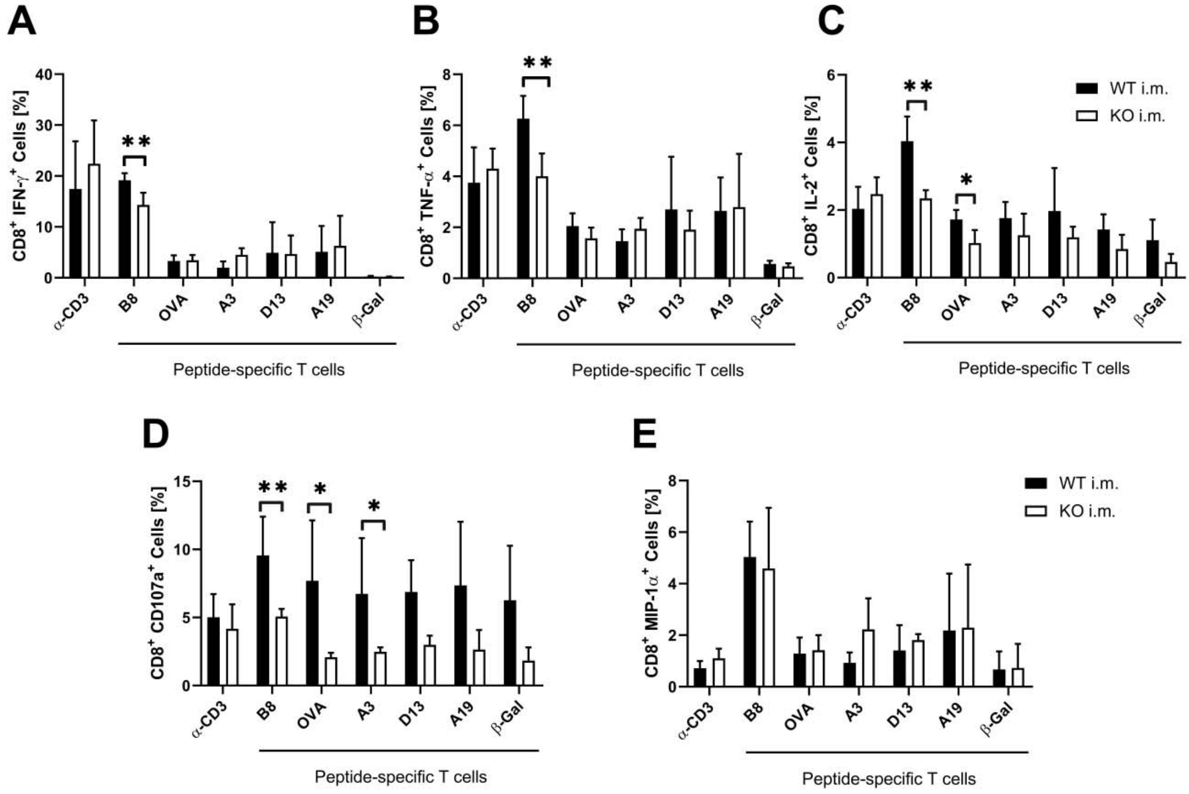

Supplementary Figure 2: STING has a strong impact on shaping the immunodominance of MVA-specific immune responses after i. m. immunization. STING KO mice (STING KO) and WT-littermates (WT) were vaccinated on day 0 i. m. with 10^7 IU MVA-P7.5-OVA. Seven days after priming antigen-specific $CD8^+$ T cells were analyzed *ex vivo* by flow cytometry for their functionality by expression of (**A**) IFN-γ, (**B**) TNF-α, (**C**) IL-2, (**D**) CD107a and (**E**) MIP-1α. Data are represented as mean ± SD of n=5 mice per group pooled from two independent experiments. Statistical significance (*P*); * = $P \leq 0.05$; ** = $P \leq 0.01$.

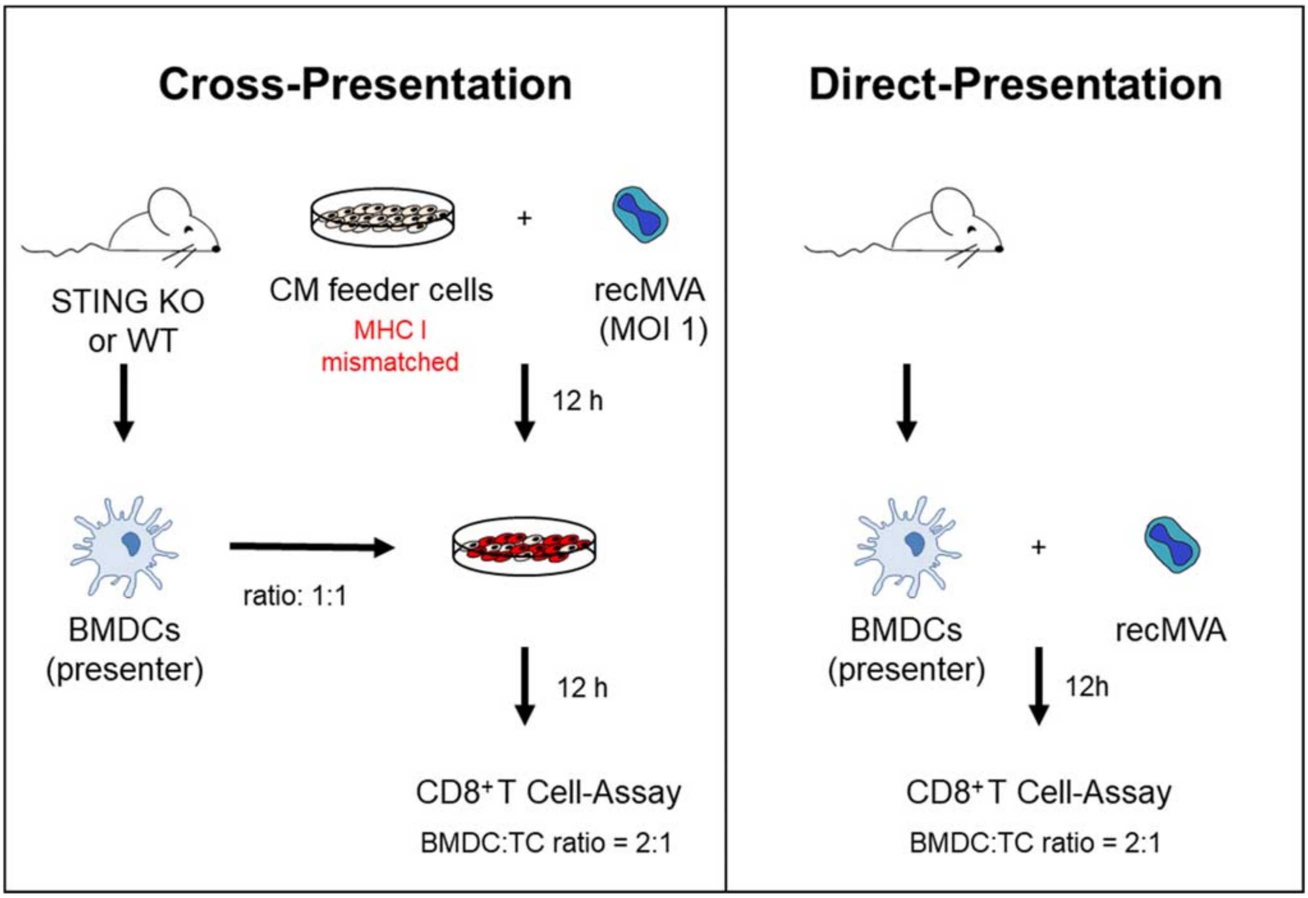

Supplementary Figure 3: Cross- and direct-presentation assay. For **cross-presentation**, MHC I-mismatched feeder cells were infected with recombinant MVA (recMVA) at MOI 1 for 12 h. After washing and virus inactivation by PUVA treatment, infected Cloudman S91 melanoma (CM) feeder cells expressing STING (WT) or not (STING KO) were co-cultured with bone marrow-derived dendritic cells (BMDCs) derived from either STING KO mice or WT-littermates for 12 h. For **direct-presentation**, BMDCs derived from STING KO mice or WT-littermates were infected with MVA for 12 h. In both settings, BMDCs were incubated with $CD8^+$ T cell (TC) lines for 4 h after washing. As read-out for the antigen presentation capability of cross- or directly presenting BMDCs, T cell activation of peptide-specific $CD8^+$ T cells was determined by IFN-γ production (intracellular cytokine staining (ICS) followed by FACS analysis).

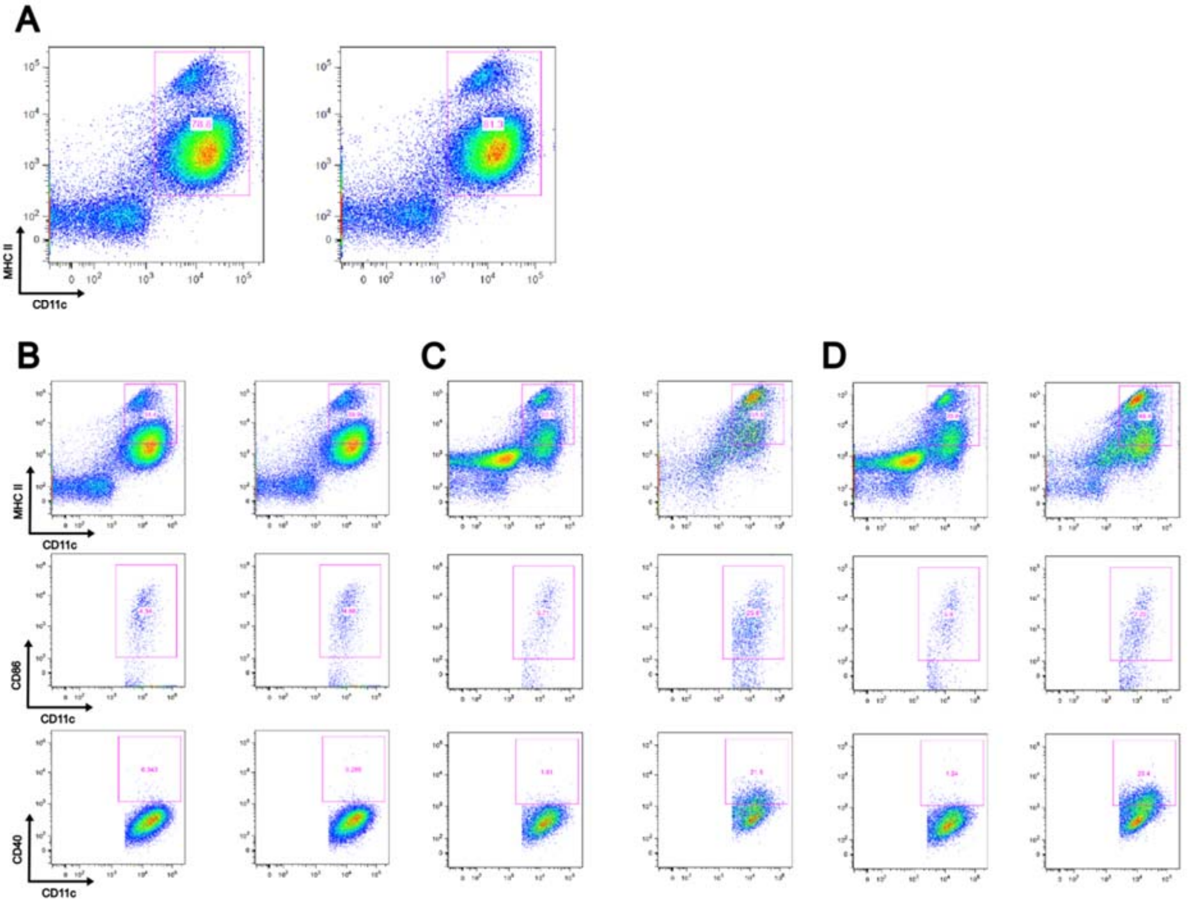

Supplementary Figure 4: Purity of BMDC cultures and expression of maturation markers by STING KO and WT BMDCs. (A) Purity of BMDC cultures derived from WT (left) or STING KO mice (right) were analyzed on day 7 after treatment with GM-CSF for the expression of CD11c by FACS after exclusion of dead cells using a viability dye. Maturation status of BMDCs (**B**) at day 7 of GM-CSF culture prior to experimental use (infection or co-culturing with feeder cells) or (**C, D**) subsequently after co-culturing with feeder cells in antigen presentation assays. (**B**) WT (right) and STING KO BMDCs (left) were analyzed for MHC II (top), CD86 (middle) and CD40 expression (bottom) prior to experiments. Alternatively, (**C**) WT BMDCs or (**D**) STING KO BMDCs were co-cultured with (left) mock-infected or (right) MVA-infected wildtype feeder cells and maturation marker expression was analyzed 20 h post co-culture.

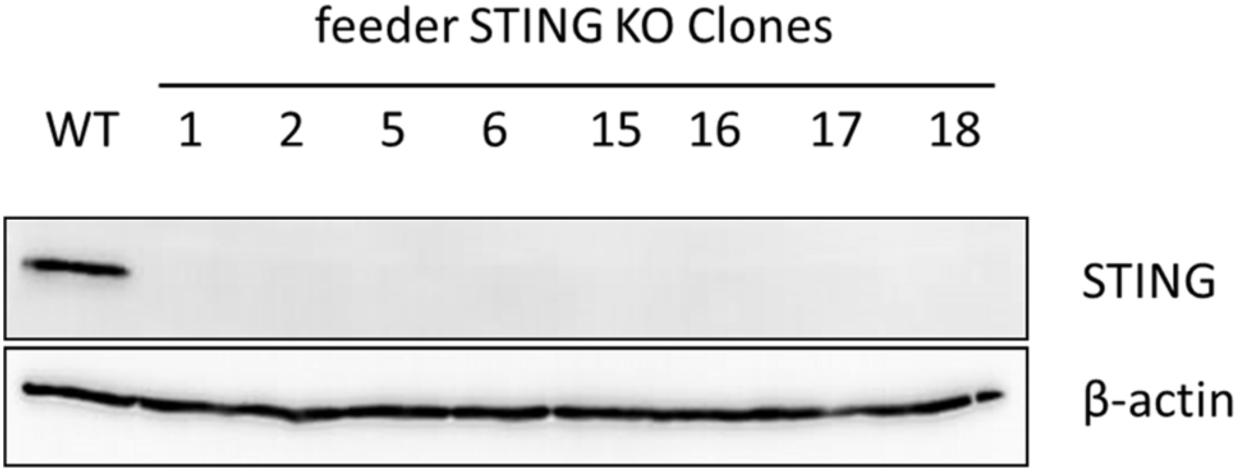

Supplementary Figure 5: Confirmation of the STING knockout in feeder cells by western blot analysis. WT feeder cells (Cloudman S91 melanoma cells) were subjected to gene-editing using CRISPR/Cas9. After puromycin selection, the bulk population was separated into single-cell clones. After expansion of clones, the absence of STING was confirmed by western blot analysis. All feeder STING KO clones (clone 1, 2, 5, 6, 15, 16, 17 and 18) showed deficient STING expression in comparison to control feeder cells (WT). β-actin in STING KO or WT feeder cell clones served as a loading control.

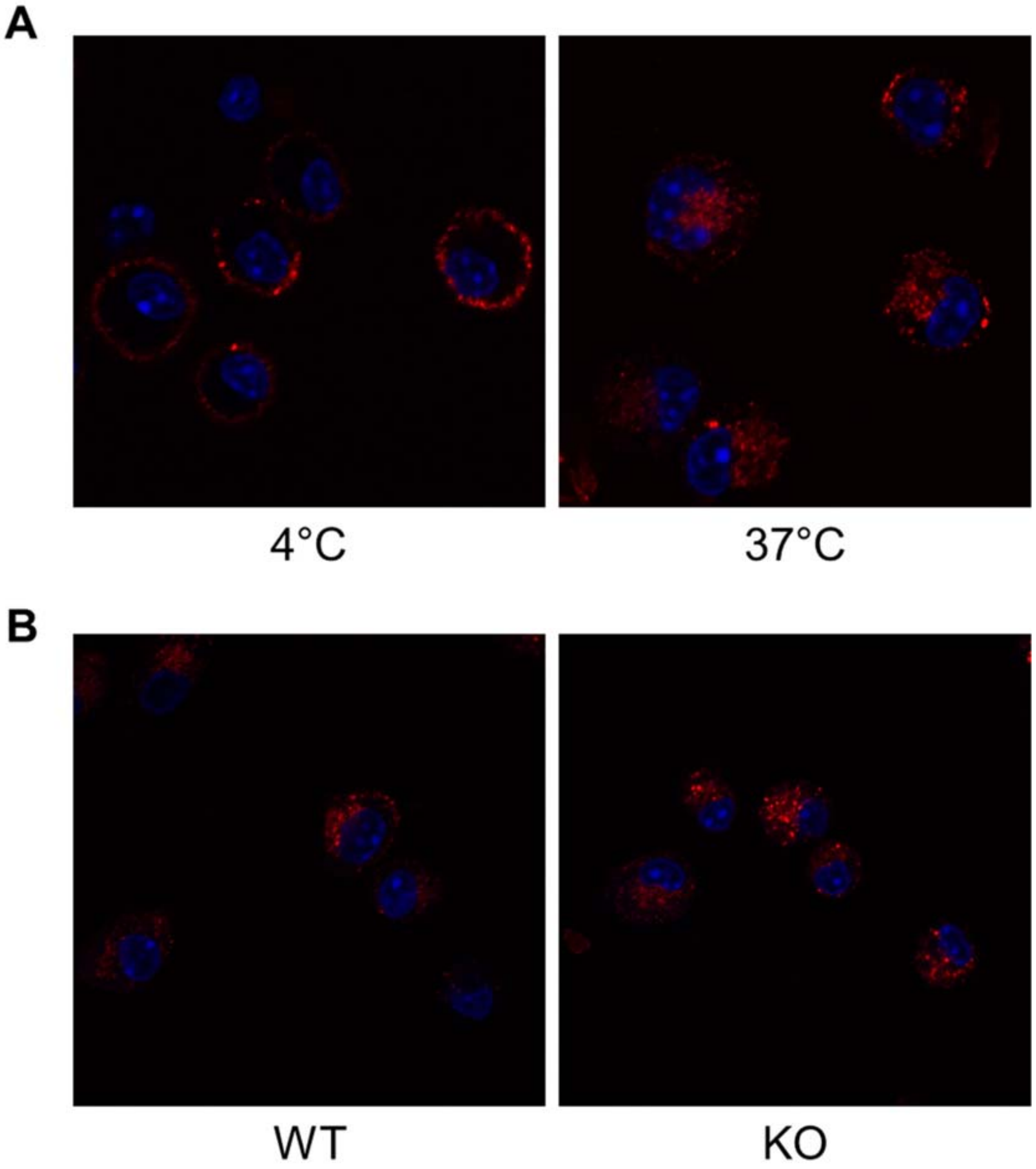

Supplementary Figure 6: STING KO and WT BMDCs showed a comparable phagocytosis activity. GM-CSF-BMDCs were generated from STING KO (KO) mice or wildtype littermates (WT). Phagocytosis activity was analyzed using DQ-OVA. (**A**) Phagocytosis activity is blocked at 4°C. WT BMDCs (WT) were incubated with 500 mg/ml DQ-OVA for 2 h at 4 or 37 °C. (**B**) STING KO (KO) and WT BMDCs (WT) were incubated with 250 mg/ml DQ-OVA for 2 h at 37 °C to determine the phagocytic activity. The fluorescence emission maximum was measured at 515 nm after excitation 505 nm.

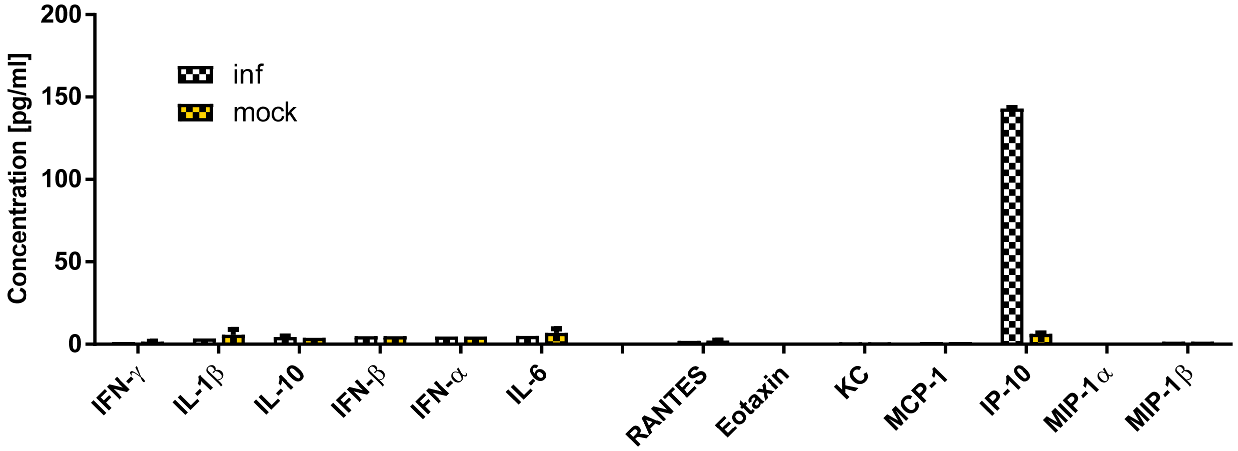

Supplementary Figure 7: Cytokine and chemokine production by MVA-infected feeder cells. WT feeder cells (Cloudman S91 melanoma cells) were infected with MVA-PK1L-OVA at MOI 1 (inf) or mock infected (mock). Supernatants were collected at 12 h post infection and concentrations of cytokines (IFN-γ, IL-1β, IL-10, IFN-β, IFN-α and IL-6) and chemokines (RANTES, Eotaxin, KC, MCP-1, IP-10, MIP-1α and MIP-1β) determined by Cytoplex assay. Data are depicted as the mean ± SD of n=2 supernatant preparations from individual cell cultures per group pooled of from two independent experiments.
